# The Prevalence and Impact of Clinical Pharmacists’ Intervention on Drug-Related Problems in Patients With Chronic Kidney Disease

**DOI:** 10.7759/cureus.59402

**Published:** 2024-04-30

**Authors:** Khurshid Alam, Amer H Hayat, Amir Ullah, Syed Azhar Syed Sulaiman, Waqas Ahmad, Guat See Ooi

**Affiliations:** 1 Clinical Pharmacy, Universiti Sains Malaysia, Gelugor, MYS; 2 Nephrology, District Head Quarter (DHQ) Hospital Medical Teaching Institution (MTI) Bannu, Bannu, PAK; 3 Pharmaceutical Chemistry, Universiti Sains Malaysia, Gelugor, MYS; 4 Pharmacy, Universiti Sains Malaysia, Gelugor, MYS

**Keywords:** drugs-related problems, poor compliance, chronic kidney disease (ckd), failure to receive drugs, drug-drug interaction, improper drug selection, prevalence

## Abstract

Chronic kidney disease (CKD) is a global health issue of growing concern. According to projections from the Worldwide Health Observatory, it is currently one of the rapidly increasing contributors to global mortality. The prevalence of CKD and end-stage renal disease (ESRD) is increasing globally. The objective was to evaluate the prevalence and impact of clinical pharmacist intervention in resolving drug-related problems (DRPs) among patients with CKD. A single-arm, pre- and post-intervention study design was used, which was assessed to be suitable in testing for the feasibility of the implementation of an intervention in clinical practice. With this study pre- and post-intervention variables of interest were measured before and after an intervention in the same patients to evaluate the impact of clinical pharmacists on ambulatory patients with CKD. The findings of this study indicate a high prevalence of DRPs, with every patient experiencing at least one DRP. The mean DRP per patient was found to be 2.903 with STD ± 1.148. The study assessed the considerable influence of clinical pharmacist intervention on DRPs. The predominant form of DRP was drug interaction 167 (45.1%) which was reduced to 76 (20.5%) after intervention carried out by clinical pharmacists statistically significant (p = 0.032). Another common DRP was found to be poor compliance issues in pre-interventions (n = 144 (38.9%)) and was reduced to 80 (21.6%) at post-intervention significantly (p = 0.042). Untreated indications were noticed in 137 cases (37.0%), after pharmacist intervention, this number was significantly reduced to 27 cases (7.3%), with a statistically significant difference (p = 0.004). However, it is noteworthy that medication compliance among patients in our study was unsatisfactory and fell below expectations. As a clinical pharmacist played an important role in reducing the prevalence of poor medication adherence to lower levels in these CKD outpatients. This research emphasizes the vital role of clinical pharmacists in mitigating DRPs among CKD patients, resulting in improved medication management and potentially better health outcomes.

## Introduction

Chronic kidney disease (CKD) is a worldwide health problem. According to a prognosis from the Worldwide Health Observatory, at present it is one of the fast-rising causes of mortality globally [1]. Throughout the world, CKD affects patients. The definition of CKD is abnormalities of kidney structure or function with health implications, with a duration of three months or more [2]. CKD is a progressive and irreversible condition, defined as an estimated glomerular filtration rate (GFR) of <60 mL/min/1.73 m^2^ and/or kidney damage (hematuria or proteinuria) present for at least three months [3].

The burden of CKD is anticipated to undergo a continual surge on a global scale due to the escalating prevalence of diabetes, which stands as the leading cause of CKD worldwide. This growing burden is expected to be particularly prominent in Asia, where the region houses more than 4.5 billion people, comprising 60% of the global population [4].

Drugs are therapeutic agents used to treat, prevent, or detect diseases or symptoms. When used incorrectly, they can cause possibly life-threatening issues, lowering quality of life and increasing morbidity or even mortality. While drug-related problems (DRPs) pose a noteworthy challenge, a significant proportion of these issues can be prevented through the identification of the root causes of medication errors and their contributing variables [5].

The definition of a DRP has been given as “an event or circumstance involving drug therapy that actually or potentially interferes with desired health outcomes” [6]. CKD has implications not only on an individual's health but also on their social life and output. The harmful impacts of CKD extend beyond individual health to society as a whole [6].

It is believed that the higher prevalence of hypertension, diabetes, and smoking among the younger population in Pakistan is contributing to a higher incidence of CKD in this age group [7]. According to a recent study, the incidence of CKD is greater in individuals under the age of 50 in Pakistan, and the leading causes of CKD include glomerulonephritis, diabetic nephropathy, renal stones, and hypertension [8]. However, more research is needed to evaluate both these aspects since there is insufficient reliable evidence.

As in the Pakistani context, the assessment of the influence of clinical pharmacists in mitigating DRPs was conducted through a prospective, interventional study implemented at a hospital associated with teaching activities. The primary objective of the study was to discern DRPs and their root causes. Out of the 161 recommendations proposed by clinical pharmacists, 86.33% (n = 139) demonstrated efficacy in rectifying the identified issues, whereas 6.83% (n = 11) of the recommendations were deemed ineffective, as they inadequately addressed the respective problems. The findings of this study provide evidence that clinical pharmacists play a crucial role in hospital activities and can significantly enhance the quality of medication use and patient safety. Furthermore, the study highlights the high acceptance of clinical pharmacist intervention by prescribers and demonstrates the positive contribution of clinical pharmacists in identifying and addressing DRPs in the healthcare setting of Pakistan [9]. The high incidence of CKD in young adults in Pakistan is likely due to the high prevalence of risk factors such as diabetes mellitus, hypertension, and renal calculi, as indicated by the results of our study [7].

CKD is on the rise in South Asian nations such as Pakistan, and the causes are multifaceted. Most people have insufficient health-care provision due to a lack of health education, a lack of primary healthcare, insufficient government funding, and, most significantly, the increasing prevalence of CKD risk factors such as diabetes and hypertension. Other reasons, such as glomerulonephritis and renal stones, are also common as a result of infections and dry weather [10].

Research regarding CKD in Pakistan emphasizes the critical need for comprehensive research and the involvement of clinical pharmacists in addressing the challenges posed by CKD. There have been segregated studies on CKD in Pakistan, but still, there's a lack of comprehensive research that provides a holistic view of the CKD burden in the country. This knowledge gap inhibits the formulation of effective strategies to address CKD-related issues [11]. The absence of comprehensive CKD research poses a challenge for public health leaders and policymakers. Without a complete understanding of the CKD burden, it's difficult to develop and implement effective strategies to reduce CKD-related mortality and morbidity [11].

In developing countries like Pakistan, clinical pharmacists play a crucial role in bridging the gap in healthcare services. Their integration can enhance the quality and safety of care, optimize medication use, and ultimately improve health outcomes. Given the importance of clinical pharmacists and the lack of comprehensive CKD research, there's a pressing need for additional research focused on investigating the prevalence and clinical significance of DRPs in CKD patients in Pakistan.

## Materials and methods

Study design

This was a single-arm, pre- and post-intervention study design, which was assessed to be suitable in testing for the feasibility of the implementation of an intervention in clinical practice [12]. With this study pre- and post-intervention variables of interest were measured before and after an intervention in the same patients to evaluate the impact of clinical pharmacists on ambulatory patients with CKD. The study had two phases; the first phase was a pre-intervention phase. It was an observational prospective phase in which a simple random sample of patients attending the outpatient nephrology department was chosen as a pre-intervention group, that was to have baseline information to enable the assessment of whether the impact of pharmacists’ intervention would result in any change in the DRPs and other outcomes. The second phase was the intervention phase in which the pharmacists carried out the services on DRPs and other outcomes services and relevant data were collected.

Study population

The study population consisted of adult patients diagnosed with CKD stages 1 to 5 and undergoing treatment and follow-up at Khyber Teaching Hospital (KTH), Peshawar, Pakistan, and District Head Quarter (DHQ) Hospital Medical Teaching Institution (MTI) Bannu, Bannu, Pakistan. Researcher pharmacists performed interventions on DRPs in CKD patients prescribing for outpatients at the outpatient nephrology department.

Eligibility criteria

Inclusion Criteria

Patients included were adults aged 18 years and older diagnosed with CKD, receiving treatment at the outpatient nephrology department, possessing comprehensive medical records, prescribing at least one medication as part of their treatment regimen, and expressing willingness to cooperate with clinical pharmacists for medication reconciliation.

Exclusion Criteria

Excluded patients were those undergoing dialysis treatment, individuals diagnosed with acute kidney disease, pregnant patients, those with cognitive impairments, minors under the age of 18, patients with incomplete medical records, individuals not prescribed any medication for CKD management, and patients declining to participate in medication reconciliation efforts alongside clinical pharmacists.

Study location and time

The study was conducted at the outpatient nephrology department of Khyber Teaching Hospital (KTH), Khyber Pakhtunkhwa (KPK), and District Head Quarter Hospital Bannu, Bannu, Pakistan, a relatively low-income major city in north-Western Pakistan with a youth and ethnically homogeneous population. Peshawar is Pakistan's sixth most populous metropolis, with a population of over 2.3 million people. The duration of the study was from February 2022 to June 2022, and then from March 2022 to July 2022.

The outpatient nephrology department provides care to patients from all over the KPK province. The team of prescribers consists of consultant nephrologists, a nephrologist, a clinical specialist training to become a nephrologist, medical officers, and house officers. The nephrology department is highly crowded and most of the time the patients are more in number and they do not gain the appropriate time to be examined by nephrologists properly. As there is lacking of medical, clinical staff, and pharmacists.

Sampling technique

For data collection in this study, a simple random sampling method was used. A simple random sample is a method of selecting a subset of individuals from a larger population, where each member of the population has an equal chance of being chosen. At the beginning of the nephrology outpatient department, a list of patients who had appointments was obtained from the director in charge of the nephrology. This list served as the sampling frame, and participants were randomly selected from it to be included in the study. Simple randomization was carried out based on patients’ medical record numbers (MRN), we utilized the RANDBETWEEN function, which generates random integers between a specified lower and upper bound. In our study, we generated a random number between 1 and 3000 whereby every second patient was recruited for the study.

Sample size calculation

DRPs were one of the main measure outcomes of this study. Sample size calculation for categorical data is based on proportions. From a literature review of previous studies from other parts of the world in this field, the proportion before intervention was estimated to be around 50%, and the researchers were hoping to be able to decrease this to half (25%). For significant level (=0.01) and power β = 0.09, the required sample size from the nomogram was 160 patients [13]. To justify potential dropouts’ possibility, we increased the sample size, used in our calculations by incorporating extra patients, so the researchers decided to have a larger sample, 370 subjects were included in the study in each pre- and post-group. It represented around 50% of the patients who visited the outpatient nephrology department during the study time (population).

For the recruitment of the patients, strategies were used such as collaboration with Nephrologists to work closely on the patients of CKD and healthcare providers to identify eligible patients according to the inclusion and exclusion criteria during routine medical examination of the patients at outpatient nephrology department. Also, personalized approaches, such as phone calls, were used to directly contact eligible CKD patients and invite them to participate. Ensured a clear and comprehensive consent process, providing sufficient opportunity for CKD patients to ask questions and make informed decisions about participation.

Approval of the study

The study was approved by the Ethics Committee of Khyber Teaching Hospital with the registration number 160/DME/KMC.

Study procedure

Pre-intervention Phase

It was a pre- and post-study design carried out from the beginning of the study period from February 2022 to June 2022, and then from March 2022 to July 2022. A random sample of 370 patients was taken, meeting the inclusion criteria from the patients who attended the outpatient nephrology department during that period.

Data was collected from the patient’s medical records and chart review. Data included were demographics: age, gender, weight, height, body mass index (BMI), clinical condition, complications and comorbidities, reasons for visiting the nephrology, diagnostic tests and vital signs, drug therapy, their doses, dosage forms, frequency and duration, DRPs, the presence of any medication dosing error or use of a contraindicated drug.

Intervention tool development 

To perform the clinical pharmacist intervention, a structured data collection form was used and CKD patients were screened out on the basis of the inclusion criteria of my research. The structured data collection form was used to collect the relevant information from the patient and the patient's medical record and review the patient file after the patient gave the informed consent. The first part of the data collection form consisted of socio-demographic characteristics and medical history, and the second part comprised relevant clinical information and laboratory data of the patients from the medical record and medication chart reviews. The third section constituted the evaluation of DRPs including the presence, classification, and probable causes of DRPs. We utilized a selection of globally used references that are reliable and up-to-date, such as the Renal Drug Handbook: The Ultimate Prescribing Guide for Renal Practitioners, British National Formulary (BNF-58), and drug-drug interactions were evaluated by referred three sources such as Lexicomp, Medscape, and Micromedex.

Introduction of the intervention phase

The clinical pharmacist screened all patients with CKD, who visited the nephrology outpatient department based on the inclusion criteria. Initially, patients were enrolled in the pre-intervention group. Subsequently, once the pre-intervention group was completed, patients with the same condition of CKD were recruited into the intervention group. The control phase and the intervention phase took place after each other. All patients provided written informed consent.

In this study, potential DRPs were defined as pharmaceutical issues in prescriptions that were detected by pharmacists, and pharmaceutical issues and concerns identified were discussed with the nephrologist and nephrology senior medical officers. Specifically, potential DRPs were considered as DRPs of which the nephrologist was not aware, or that were not resolved until the pharmacist performed pharmaceutical intervention. We defined our original potential 12 DRP types with subtypes by referring to the Hepler-Strand classification, with modifications [14]. Pharmacists’ interventions and comments on the prescriptions were used to revise each DRP and classify it into the following categories. The service offered by the clinical pharmacist included all possible interventions that are helpful and pharmacists’ interventions and comments written on the prescriptions were used on each patient's medication chart to review and classify it into different categories. The information source, which included laboratory tests, pharmacy records, the nephrologist's prescription medication list, and information on over-the-counter (OTC) medication or natural product use, was used to describe the identified DRPs. These problems were then categorized into one of the following categories based on [14] DRP classification. These are, improper drug selection, a) prescribing the wrong drugs, b) duplication of drugs with similar effects, c) administration of stoppable drugs, d) contraindication of drugs, 1) drug interaction, 2) failure to receive medication, 3), overdose, 4) subtherapeutic, 5) poor compliance issues, 6) drugs without indication (untreated indications (UIs)), 7) under-treated indication, 8) presence of adverse drug reactions.

At pre-intervention phase

To conduct a thorough medication review and address DRPs in outpatient settings, a systematic approach was implemented. This involved systematically collecting and interpreting relevant information regarding the patient's medical history, current therapeutic regimen, as well as their specific needs and concerns. During this process, a clinical pharmacist (investigator) carefully examined the patient's medical records, including their medication history, current therapeutic regimen, laboratory results, and any documented allergies or adverse drug reactions.

Subsequently, based on the comprehensive information gathered, the clinical pharmacist made appropriate recommendations within the context of the reviewed profile and other clinical data. These recommendations were then presented to the prescriber and/or other healthcare professionals involved in the patient's care, particularly in the nephrology outpatient department. The recommendations encompassed various aspects such as medication adjustments, changes in dosing or frequency, discontinuation or addition of medications, as well as instructions and counseling for the patients. The prescriber considered these recommendations in conjunction with their own clinical judgment to make well-informed decisions regarding the patient's treatment plan.

Intervention group (intervention care)

In the intervention phase, the researchers extended consultations with the nephrologist to discuss the drug-related issue and recommendations, and subsequently, patients were individual would contact and counseled on their particular disease condition and medication. After a structured medication review by the clinical pharmacist, additional drug recommendations would be proposed to the nephrologist, consultant, and trained medical officers. If the pharmacist’s and nephrologist’s assessments of clinical relevance agreed, then would proceed with the patient’s prescription medications. Patients had the opportunity to ask medication-related questions and were offered advice about individual medication-related problems such as compliance, drug-drug interaction, frequency issues such as administration problems or adverse drug reactions. The clinical pharmacist provided a full spectrum of potentially useful interventions, encompassing tasks like conducting audits and providing feedback, issuing reminders, and engaging in discussions with every prescriber individually and so, the clinical pharmacist would engage with the CKD patients and the prescribers from 9 am to 2 pm in the outpatient nephrology department for the management of DRPs and other concerns that were inappropriate according to the medical condition of the patients particularly according to the creatinine clearance, stages of the CKD. This allowed the clinical pharmacist to review the patient’s medical records and medical charts and to write down detailed notes. The clinical pharmacist communicated his clinical interventions and recommendations to the respective nephrologists and prescribers and documented the response. The clinical pharmacist would document the record of whether the recommendations were approved, modified, or rejected. The outcomes of the study depended on the prescribers’ acceptance of the pharmacist’s recommendations. The prescribers were completely free to accept or reject the pharmaceutical interventions and recommendations. Clinical pharmacists performed interventions on DRPs in prescribing for CKD outpatients at the nephrology department. Clinical pharmacists would discuss each individual case with the nephrology doctors on DRPs’ determination results in the pre-intervention phase for 15 to 20 minutes. Clinical pharmacists discussed this with each nephrologist, consultant, or senior nephrology resident twice a week during the intervention period from February 2022 to July 2022.

Outcomes measurement

The outcomes measured in this study were the impact of clinical pharmacists' intervention in resolving various DRPs, pre- and post-pharmacist involvement. These DRPs were categorized based on (Hepler & Strand, 1990) DRP classification. These are improper drug selection, a) prescribing the wrong drugs, b) duplication of drugs with similar effects, c) administration of stoppable drugs, d)contraindication of drugs, 1) drug interaction, 2) failure to receive medication, 3) overdose, 4) subtherapeutic, 5) poor compliance issues, 6) drugs without indication (UIs) 7) under-treated indication, 8) presence of adverse drug reactions.

Variables and operational definition

The primary outcome was DRPs in these patients. DRP was defined as an event or circumstance that involves a patient’s drug treatment that actually, or potentially, interferes with the achievement of an optimal outcome [14].

Improper drug selection

An improper drug selection (IDS) is a situation in which the patient has been prescribed the wrong drug [14].

Drug interactions

Drug interactions (DIs), result from drug-drug, drug-food, and drug-laboratory interactions (14). They can occur in patients receiving drugs from different pharmacological classes as well as within the same pharmacological class.

Failure to receive the drug

Failure to receive the drug (FRD) means the patient is not receiving prescribed medications for a given medical condition [14]. Patient non-adherence or non-compliance was considered when examining instances of failure to receive the prescribed medication.

Overdose

As stated by Cipolle et al. [15], when a patient receives a dose of an agent that is too high and experiences a dose-dependent or concentration-dependent toxic effect, he or she is experiencing a DTP. In patients with decreased renal function, the ability of the kidney to eliminate drugs and their metabolites is decreased, which in turn leads to the accumulation of drugs and toxic products in the kidney. Overdose is defined as when the patient is taking too much of the correct medication for a given medical condition [14].

Subtherapeutic dosage

Subtherapeutic dosage (STD) means the patient is taking too little of the correct drug for a given medical condition [14]. STD was identified where the frequency of dosing was lower than recommended. An example was a once-daily dosing of Metoprolol instead of the recommended twice-daily dosing.

Poor compliance issues

A patient’s non-adherence to a drug regimen can be defined as the patient’s inability or unwillingness to follow a drug regimen that has been prescribed by the practitioner and judged to be clinically appropriate, effective, and able to produce the desired outcome without harmful effects. Non-adherence can be due to a number of reasons. Some are within the patient’s control and some are beyond it [14].

Untreated indications

UIs means that the patient has a medical problem that requires drug therapy (an indication for drug use) but is not receiving a drug for that indication [14]. This study included patient diagnoses that were not being addressed.

Drug without indication

Drug without indication (DWI) means the patient has no valid medical condition for taking a certain drug [14]. A DWI occurs when a patient takes an unnecessary drug therapy for which the clinical indication is not present at that time [16].

Presence of adverse drug reactions 

Adverse drug reaction/effect (ADR) means that unwanted/unpleasant or harmful drug effects cause a medical condition in a patient who used the normal dose of the drug for a normal medical condition [14]. As stated by Cipolle et al. [16], “ADRs can be defined as undesirable negative effects caused by the medication that was not predictable based on its dosage concentration or pharmacological action.”

Data analysis

Statistical data analysis was performed using the IBM SPSS Statistics for Windows, Version 26 (Released 2019; IBM Corp., Armonk, New York, United States). The sociodemographic and clinical characteristics of patients with CKD were presented using descriptive statistics such as means and percentages. Categorical variables were articulated as numbers and percentages; continuous variables were expressed as mean with standard deviation. As most of the data were categorical data with "yes" or "no" questions, crosstabulation with a chi-square test was used to compare the pre-intervention and post-intervention patients' DRPs. Fisher’s exact test was used in analyzing 2 x 2 tables with an expected cell frequency of less than five. A two-sided p-value < 0.05 was considered statistically significant.

## Results

Sociodemographic characteristics

By recognizing the DRPs among CKD patients, nephrologists and healthcare providers can implement strategies to minimize risks and improve patient care and outcomes. A total of 740 patients were randomly selected with 370 patients in the pre-intervention phase and 370 patients in the intervention phase. The demographic and clinical characteristics of the patients presented here include gender, age, education, employment status, monthly income, marital status, stage of CKD, smoking habits, family history of CKD, comorbidities and stages of CKD, serum creatinine, and number of DRPs per patient as illustrated in Table 1.

**Table 1:** Sociodemographic characteristics CKD: chronic kidney disease; DRPs: drug-related problems

Variables	Frequency (%)
Gender	
Female	144 (38.9)
Male	226 (61.1)
Age, mean ± SD	40.85 ± 15.69
Education	
No formal education	96 (25.9)
Primary	139 (37.6)
Secondary	90 (24.3)
Matriculation	1 (0.3)
College	8 (2.2)
University level	31 (8.4)
Diploma	5 (1.4)
Employment status	
Government employee	90 (24.3)
Unemployed	71 (19.2)
Self-employed	27 (7.3)
Household	86 (23.2)
Retired	57 (15.4)
private	39 (10.5)
Monthly income (PKR)	
<10K	259 (70.0)
10-30K	38 (10.3)
30-50K	21 (5.7)
50-100K	51 (13.8)
100K-200K	1 (0.3)
Marital status	
Married	364 (98.4)
Single	2 (0.5)
Widowed	4 (1.1)
Smoking habit	
Smoker	28 (7.6)
Non-smoker	342 (92.4)
Family history of CKD	70 (18.9)
Comorbidities	
Hypertension history	333 (90.0)
Uncontrolled hypertension (>140/90)	269 (72.7)/175(47.3)
Hypertension (<140/90)	208(56.2)/195(52.7)
Diabetes mellitus history	196 (53.0)
Chronic glomerulonephritis history	20 (5.4)
Hyperlipidemia history	11 (3.0)
Obstructive uropathy history	100 (27.0)
Pylonephritis history	54 (14.6)
Dehyration history	83 (22.4)
Malnutrition history	12 (3.2)
Stage of chronic kidney disease (Estimated Glomerulus filtration (eGFR)	
Stage 2 (G-60-89 mL)	2 (0.5)
Stage 3 (G3a-45-59 mL/min)	80 (21.6)
Stage 3 (G3b-30-44 mL/min)	241 (65.1)
Stage 4 (G4-15-29 mL/min)	47 (12.7)
SCr (mg/dL)	3.53 ± 1.62
Number of DRPs per patient mean ± SD	2.903 ± 1.148

Comorbidities history

The duration of hypertension of 6 to 10 years was noticed highest among 209 (56.5%) patients, while the lowest was observed in 8 (2.2%). The comorbidities history of hypertension and diabetes are shown in Table 2.

**Table 2:** Duration of comorbidities history

Comorbidities	
Hypertension	
Duration not known	20 (5.4)
<5 years	128 (34.6)
6-10 years	209 (56.5)
11-15 years	5 (1.4)
16-20 years	8 (2.2)
Diabetes	
Duration not known	84 (22.7)
<5 years	225 (60.8)
6-10 years	55 (14.9)
11-15 years	2 (0.5)
16-20 years	3 (0.8)
>20 years	1 (0.3)

Prevalence of DRPs among CKD patients

Table 3 below provides a summary of the occurrence rates of different DRPs among patients with CKD before and after pharmacist intervention. The results showed a substantial reduction in the prevalence of DRPs after the pharmacist intervention. The prevalence of "wrong drug prescription" decreased from 23.5% to 5.1%, "drug with similar effect" decreased from 32.7% to 17.8%, and "administration stoppable drugs" decreased from 33.0% to 15.4%. The prevalence of "contraindication of drugs" decreased from 22.7% to 15.7%, and "drug interaction" decreased from 45.1% to 20.5%.

**Table 3:** Prevalence of dug related problems among CKD patients CKD: chronic kidney disease; DRPs: drug-related problems

DRPs	Pre-intervention n = 370 N (%)	Post-intervention n = 370 N (%)
Improper drug selection (a, b, c, d)		
a. Prescribing the wrong drug	87 (23.5)	19 (5.1)
b. Duplication of drug with similar effect	121 (32.7%)	66 (17.8%)
c. Administration of stoppable drug	122 (33.0)	57 (15.4)
d. Contraindication of drugs	84 (22.7)	58 (15.7)
Drug interaction	167 (45.1%)	76(20.5%)
Failure to receive drugs	135 (36.5%)	66 (17.8%)
Overdose	73 (19.7%)	8 (2.2%)
Subtherapeutic	66 (17.8%)	15(4.1%)
Compliance issues	144 (38.9%)	80 (21.6%)
Untreated indication	137 (37.0%)	27(7.3%)
Drugs without indication (under-treated indication)	116 (31.4%)	44 (11.9)
Presence of adverse drug reactions	90 (24.3%)	13 (3.5%)

The pharmacist intervention also led to a decrease in the prevalence of "failure to receive drugs" from 36.5% to 17.8%, "overdose" from 19.7% to 2.2%, and "subtherapeutic" from 17.8% to 4.1%. The prevalence of "compliance issues" decreased from 38.9% to 21.6%, "UI" decreased from 37.0% to 7.3%, and "under-treated indication" decreased from 31.4% to 11.9%. Furthermore, the incidences of adverse drug reactions decreased from 24.3% to 3.5%, respectively, in Table 3.

The impact of clinical pharmacists’ intervention on DRPs among CKD patients at pre- and post-intervention levels (Table 4).

**Table 4:** The impact of clinical pharmacist intervention on drug-related problems among CKD patients at the outpatient nephrology department *: Chi-square; **: Fisher’s exact test CKD: chronic kidney disease

DRPs	Total n = 740 N (%)	Pre-intervention n = 370 N (%)	Post-intervention n = 370 N (%)	p-value
Improper drug selection (a, b, c, d)				
a. Prescribing the wrong drug	106 (14.3)	87 (23.5)	19 (5.1)	0.055**
b. Duplication of drug with similar effect	187 (25.4)	121 (32.7)	66 (17.8)	0.001*
c. Administration of stoppable drug	179 (24.1)	122 (33.0)	57 (15.4)	0.005*
d. Contraindication of drugs	142 (19.1)	84 (22.0)	58(15.7)	0.020*
Drug interaction	243 (32.8)	167 (45.1)	76(20.5)	0.032
Failure to receive medication	201 (27.1)	135 (36.5)	66 (17.8)	0.012*
Overdose	81(10.9)	73 (19.7)	8 (2.2)	0.052**
Subtherapeutic	81 (10.9)	66 (17.8)	15(4.1)	0.110*
Poor compliance issues	224 (30.2)	144 (38.9)	80 (21.6)	0.042*
Untreated indications	154 (22.1)	137(37.0)	27 (7.3)	0.004
Drugs without indication (under-treated indication)	160 (21.6)	116 (31.4)	44 (11.9)	0.072*
Presence of adverse drug reactions	103 (13.9)	90 (24.3)	13 (3.5)	0.062*

The table presents data on DRPs and their distribution before and after performing an intervention. The overall incidence of improper drug selection decreased from pre-intervention to post-intervention. Prescribing the wrong drug showed a statistically significant (p = 0.001) decrease from 121 (32.7%) to 66 (17.8%). The occurrence of duplicating drugs with a similar effect exhibited a notable decrease, declining significantly from 121 (32.7%) to 66 (17.8%) with a p-value of 0.020. Similarly, there was a significant reduction in the administration of stoppable drugs, decreasing from 121 (32.7%) to 66 (17.8%) with p = 0.005. Contradiction of drugs showed a significant decrease (p = 0.042) from 84 (22.0%) to 58 (15.7%). The incidence of drug interactions decreased significantly (p = 0.032) from 167 (45.1%) to 76 (20.5%) at post-intervention.

The incidence of failure to receive medication decreased significantly (p = 0.012) from 135 (36.5) to 66 (17.8), after the intervention. Overdose cases showed a decreasing trend but the difference was not statistically significant. STD showed a decreasing trend but the difference was not statistically significant. The incidence of poor compliance issues decreased significantly post-intervention from 144 (38.9%) to 80 (21.6%) (p = 0.042). UIs significantly (p = 0.004) decreased from 137 (37.0%) to 27 (7.3%) after the intervention. Drugs without indication (under-treated indication) showed a decreasing trend but the difference was not statistically significant. The presence of adverse drug reactions showed a decreasing trend, but the difference was not statistically significant as illustrated in Table 4.

## Discussion

Prevalence of DRPs

In this study, we have found a high level of DRPs. Also, each patient had at least one DRP, and the mean DRP per patient was found to be 2.903 with STD ± 1.148 and the DRP per patient ranges between 1 and 7. In a previous study that reported DRPs, 87 patients (77%) identified 101 DRPs (the average DRP number per patient was 1.16 [17]. According to a study performed by Roy et al. [18], a total of 723 instances of DRPs were detected. Most of them were potential problems, and on average, patients had 3.61 DRPs, which was significantly lower than the 10 DRPs per patient reported in the research conducted by Ramadaniati et al. [19]. The discrepancy between these findings can be attributed to clinical pharmacists' prompt identification and reporting of DRPs to the relevant medical professionals. Clinical pharmacists play a vital role in identifying and addressing DRPs that may arise during patient care. They work closely with other healthcare professionals to ensure that patients receive optimal medication therapy. By detecting and reporting DRPs to relevant healthcare providers, clinical pharmacists can help prevent adverse drug events and optimize patient outcomes. Clinical pharmacists can also play a vital role in providing medication education to patients and caregivers, ensuring that patients understand the proper use of their medications and potential side effects. The increased incidence of DRPs in the former study may be attributed to the inclusion of inpatients with CKD, who generally have more complex clinical conditions and are often receiving dialysis, unlike the Canadian study that only included outpatients with CKD stages 3-4.

Impact of clinical pharmacists' intervention on DRPs

Identifying the contributing factors for DRPs is of utmost importance as it enables the identification of patients who are particularly susceptible and require diligent monitoring of their prescription regimens. Additionally, it facilitates the implementation of pharmaceutical care interventions. The findings of a study indicated that various demographic and clinical characteristics, including drug source, alcohol usage, number of diseases, number of drugs, and length of hospitalization, significantly influenced the occurrence of DRPs [20].

DRPs pose a significant burden on the healthcare system, causing substantial costs and complexities. These issues are frequently encountered among individuals with chronic conditions, such as diabetes patients [21]. Failure to address drug therapy problems can result in the development of clinical complications. In this study, one of the primary goals was to investigate the impact of clinical pharmacists on addressing DRPs in individuals, diagnosed with CKD. During the study period, a cohort comprising 370 CKD patients who were receiving outpatient care in the nephrology department were actively engaged in pharmaceutical interventions led by clinical pharmacists.

Improper drug selection

One of the primary objectives of this study was to evaluate, comprehend, and address the potential DRPs among patients with CKD, emphasizing the significant role of pharmaceutical interventions in clinical practice. The study specifically focused on addressing the discontinuation or substitution of drugs that presented DRPs for CKD patients. Special consideration was given to patients with elevated blood pressure, diabetes mellitus, BMI, different stages of CKD, complex medication regimens combined with comorbidities, and other complications. These factors were identified as contributing to a higher risk of treatment-related drug problems.

These specific DRPs required the intervention of pharmacists to address the issues that nephrology specialists or nephrology residents were either unaware of or had been unable to resolve prior to pharmacist intervention. We adapted our original potential nine DRP types and a few subtypes by referring to the Hepler-Strand classification, by incorporating modifications.

The DRPs that were identified were characterized based on the information sources available, including laboratory tests, pharmacy records, the nephrologist's prescription medication list, and information regarding the use of OTC medications or natural products. These problems were then assigned to one of the following categories, according to the DRP classification system developed by Strand et al. [14], 1) improper drug selection with subtypes, a) prescribing the wrong drug, b) duplication of drugs with similar effect, c) administration of a stoppable drug, and d) contraindication of drugs, 2) drug interaction, 3) failure to receive medication, 4) overdosage, 5) subtherapeutic, 6) poor compliance issues, 7) UIs, 8) drugs without indication, and 9) presence of adverse drug reactions. 

Following a thorough analysis of the pharmaceutical interventions, each intervention was categorized and matched to a specific potential DRP subtype as shown in Table 4.4. In our study, the most common type of DRP was found to be drug interaction 167 (45.1%) at the pre-intervention level which was reduced to 76 (20.5%) after intervention carried out by pharmacist with statistically significant (p = 0.032). Another type of DRP was poor compliance issues at the pre-intervention (n = 144 (38.9%)) stage and was reduced to 80 (21.6%) at the post-intervention level and found statistically significant (p = 0.042).

During the intervention phase, there were instances of improper drug selection and prescribing of the wrong drug, resulting in DRPs that involve specific subtypes of DRPs. In this study, we detected prescribing the wrong drug as a major potential DRP, which was found to be 87 (23.5%) in our CKD patients in the pre-intervention phase and was reduced to 19 (5.1%) in the post-intervention phase in the pharmacist incorporation, but the association was not significant (p = 0.055). The impact of a clinical pharmacist before and after the intervention was assessed in terms of the incidence of duplicate drugs with similar effects. Overall, improper drug selection representing the most frequent DRP subtype "duplication of drug with a similar effect" as a DRP was observed to be 121 (32.7%) in the pre-intervention phase while at the post-intervention phase was found to be 66 (17.8%) by the intervention of clinical pharmacist with statistically significant value (p = 0.001).

The identification of duplicative medications with similar effects and unnecessary medications was one of the notable findings in our study. For instance, we detected the concurrent prescription of proton pump inhibitors (PPIs) such as omeprazole and famotidine for similar indications, which was considered duplicative and redundant. This indicates that the involvement of a clinical pharmacist in the medication management process can be effective in reducing the occurrence of drug duplication, which is an important factor in preventing medication errors and improving patient safety.

The prevalence of dose-related DRPs in our study was lower than that reported by [22] in their study on "Treatment related problems for outpatients with chronic diseases in Jordan," which found that 50.3% of DRPs were related to inappropriate dosages according to indication and guidelines. This suggests that DRPs related to dosage may be more common in patients with chronic diseases. One plausible reason for this could be that the opportunistic diagnosis and nature of CKD may have contributed to a higher incidence of inappropriate prescribing.

Administration of stoppable drugs

Administration of stoppable drugs was observed at pre-intervention level 122 (33.0%) and at post-intervention level 57 (15.4%), which was statistically significant (p = 0.005). In this study, several specific types of stoppable drugs were identified that were commonly administered to patients. The intervention by clinical pharmacists resulted in a significant reduction in the administration of these stoppable drugs, indicating the effectiveness of the intervention in improving medication safety and appropriateness. The researcher’s intervention led to some examples such as nitrofurantoin, morphine, nonsteroidal anti-inflammatory drugs (NSAIDs), herbal medication, and glibenclamide which may be a risk for deteriorating further CKD patients and thus were discontinued. Due to safety concerns, the use of rosuvastatin was discontinued and atorvastatin was recommended as a suitable alternative for such CKD patients. These medications were considered inappropriate, possibly indicating a lack of awareness or insignificance on the part of the prescribers. It is possible that they overlooked or underestimated the potential risks associated with these medications, considering them less significant compared to the severity of the condition being treated.

This study identified a notable number of contraindicated drugs, accounting for 84 (22.0%) cases during the pre-intervention phase. However, after the intervention, the number decreased to 58 (15.7%), demonstrating a statistically significant reduction (p = 0.020). This indicates the effectiveness of the intervention in addressing and justifying the use of contraindicated medications, which highlights the importance of pharmacist intervention in minimizing the risks associated with medication that could potentially lead to adverse effects. The use of nitrofurantoin is contraindicated in cases of impaired renal function as it diminishes its effectiveness. Such comparable findings were observed in the previous studies. The commonly observed inappropriate prescriptions include NSAIDs, atenolol, gabapentin, glyburide, ranitidine, and nitrofurantoin [23]. 

According to Hanlon et al.'s research findings revealed that metformin constituted the highest percentage of contraindicated prescriptions, which can be attributed to its extensive utilization and the fact that it is not recommended for patients with an estimated glomerular filtration rate (eGFR) below 60 ml/min, as stated in the GDH guidelines [24]. The remedy for such drug problems is also suggested in a study, that approximately half of the stoppable drugs identified by pharmacists were duplicates of medications with similar effects. Consequently, interventions focusing on antiulcer drugs such as PPIs and H2 receptor antagonists were particularly effective in resolving the duplication of drugs with similar effects or duplicate prescriptions of the same drug. Enhancing collaboration between primary care physicians and community pharmacists may be necessary for future efforts to improve patient care [25].

Failure to receive medication

Within the scope of this study, one prominent DRP among CKD patients was the failure to receive medication, primarily attributed to patient non-adherence. Initially, the identified cases constituted 135 (36.5%) out of all the DRPs. However, with the intervention by clinical pharmacists, there was a significant reduction to 66 cases (17.8%) with statistical significance (p = 0.012). In our study, most of the drugs were missed by patients, such as antibiotics, antidiabetics, anti-anemic medications, and antihypertensive medications. These findings can be attributed to patients' lack of understanding regarding their disease process, the importance of adhering to prescribed medications, not the availability of the medications, and the financial implications associated with medication costs.

According to a study, non-compliance emerged as the second most commonly identified type of DRPs, accounting for 16.6% of cases, approximately 48% of non-compliance cases were attributed to the unaffordability of prescribed medications, while a lack of understanding about drug instructions was identified as another significant factor [26]. These findings can be attributed to the failure of the patients to understand their disease process and the merit of compliance with medications as prescribed. In a prior study, medication non-adherence was identified as a common DRP, with a prevalence of 16%. Patient reports revealed various reasons for medication non-adherence, including cost-related issues and the belief that symptoms had resolved [27]. This study emphasizes the importance of clinical pharmacists in CKD patient care to educate patients about their medication, their concerns, and the role that adhering to the prescribed medication can play in the healthcare system. It also explores financial assistance options, regular follow-up, and potential strategies for improving medication adherence in these kinds of patients.

Drug interaction

During the pre-intervention stage, drug interactions were identified as the most common DRPs, accounting for 167 cases (45.1%). However, after implementing clinical pharmacists’ interventions, the occurrence of drug interactions was significantly reduced to 76 cases (20.5%), with statistical significance (p = 0.032). In our study, we predominantly observed inappropriate medication combinations. For instance, the concurrent use of simvastatin and amlodipine increased the risk of myopathy, while the combination of allopurinol and ACE inhibitors like captopril enhanced the potential for toxicity. Ciprofloxacin interacts with NSAIDs such as ibuprofen and naproxen, presenting a risk of convulsions; similarly, ciprofloxacin administration with prednisolone increases the risk of tendon rupture. In some cases, the concomitant use of celecoxib with ACE inhibitors and angiotensin-II antagonists was viewed as potentially increasing the risk of nephrotoxicity and hyperkalemia, and a safe medication was recommended to resolve the risk of deterioration. Domperidone was found to be given with clarithromycin which was replaced with safe medication such as metoclopramide because of interaction with antibiotics.

The frequent occurrence of drug interactions in this context can be attributed to several factors. These include the presence of comorbidities, renal insufficiency (as in this study, the majority of the patients were in stages 3 and 4 of CKD), and the higher number of medications prescribed per patient. Furthermore, the lack of awareness and understanding among patients regarding their condition, as well as the inappropriate use of OTC drugs and substances, also contributes to the increased risk of drug interactions. Our results may resemble the findings presented in a systematic review of the utilization of multiple medications linked to adverse health outcomes, including drug interactions and poor adherence to treatment. Patients with uncontrolled blood pressure, for instance, often receive combinations of multiple drugs, which may contribute to the predisposition for DRPs. This can potentially lead to drug-drug, drug-substance interactions, or improper medication adherence by patients. Therefore, when prescribing medications that may interact with each other, healthcare providers should seriously consider the potential benefits of treatment as well as the consequences of the drug interactions, and subsequently, appropriate follow-up plans can be devised. Therefore, it would be paramount important to be vigilant in prescribing, and clinical pharmacist involvement to lessen drug interactions, particularly in patients with comorbidities like CKD. Our results closely match with those of a study conducted by Njeri et al., wherein they emphasized that the primary medication-related issues (MRPs) identified included drug interactions (22%), lack of corresponding indications for the medication (18%), and medication non-adherence (16%). Notable drug interactions observed encompassed combinations such as atorvastatin with clarithromycin, carbamazepine with rifampicin, ceftriaxone with warfarin, heparin with sulfamethoxazole, and omeprazole with warfarin [27].

Overdosage

In our study, we observed that a proportion of the medication doses in our CKD patients were identified as being too high, accounting for 73 cases (19.7%). However, after the intervention of the pharmacists, the number of cases with high medication doses was reduced to 8 (2.2%). Although the reduction was not found statistically significant (p = 0.052), the intervention of the pharmacists had a reasonable impact on optimizing the medication doses in these patients. However, we noticed some of the drugs that were overdosage according to the stages of CKD and were adjusted accordingly. The dosage of a medication often affects both its therapeutic benefits and potential side effects. Some of the examples are referred to here as, ACE inhibitors such as enalapril, allopurinol, cefuroxime and cephalosporin, meropenem, vancomycin, piperacillin/tazobactam, ciprofloxacin tramadol and dose were adjusted.

It was noticed in a study that overdosage emerged as a significant potential DRP, comprising 24.6% of all potential DRPs according to the original categorization. Consequently, analysis was conducted to explore the causes of overdosage and discovered that 59.0% of cases were linked to a decline in renal function [25].

Subtherapeutic

The prevalence of subtherapeutic drug levels was observed in 66 participants (17.8%) at the pre-intervention stage but decreased to 15 (4.15%) participants following the intervention of the clinical pharmacists. However, this reduction was not statistically significant, as indicated (p = 0.110). Despite the non-significant reduction in subtherapeutic drug levels, the intervention conducted by the clinical pharmacists as a researcher played a reasonable role in optimizing medication therapy. The identification of subtherapeutic drug levels allowed for further adjustments in dosing or alternative medication selection to achieve therapeutic efficacy. Although not statistically significant, the reduction in subtherapeutic drug levels highlights the potential impact of pharmacist interventions in improving medication outcomes for patients.

Similar findings were reported in various studies that among the different subtypes of DRPs, effectiveness-related issues (specifically, dose too low) were the second most commonly encountered. The variation observed can be attributed to differences in DRP classification, experience in primary care service, study design, sample size lack of monitoring, and misdiagnosis of patients’ present complaints.

Poor compliance

In our study, we analyzed the poor compliance among CKD patients and performed the intervention to minimize the poor compliance among these patients. About 144 (38.9%) cases were reduced to 80 (21.6%) with a significant (p = 0.042). However, it is noteworthy that medication compliance among patients in our study was unsatisfactory and fell below expectations. Clinical pharmacists played an important role in reducing the prevalence of poor medication adherence to lower levels in these CKD outpatients. In the present study, a significant proportion of 38.9% of patients did not adhere adequately to their prescribed medication regimens, highlighting the need for interventions and strategies to improve medication adherence in this population. This poor medication adherence in CKD patients may be due to many reasons, such as educational background, socioeconomic status of the patients, fear of the adverse effects, the belief that the medication will not work or the trust in the doctors, and non-availability of the medications and further rely on the herbal remedies.

A study conducted by Adem et al. discovered a strong connection between the occurrence of DRPs and two factors, such as poor adherence to medication and negative beliefs about medicine. Non-compliance-related DRPs, specifically those related to poor medication adherence and negative medication beliefs, were found to be the most prevalent sub-types in our review. This correlation may be attributed to the possibility that self-medication using different substances can lead to interactions between drugs or substances, or patients not correctly following their prescribed medication regimen [28]. In a previous study, it was observed that only 12 participants (13.79%) exhibited high medication adherence, which was lower compared to our study. The study identified various factors that contribute to poor medication adherence among CKD patients, including age, educational background, economic income, concerns about drug side effects, lack of attention to their own health condition, and limited trust in healthcare providers. However, with the active intervention of clinical pharmacists, there was a significant improvement in medical education for CKD patients, resulting in enhanced medication adherence and improved quality of life [29].

Untreated indications 

During the pre-intervention phase of our study, we observed a prevalence of 137 cases (37.0%). However, following pharmacist intervention, this number was significantly reduced to 27 cases (7.3%), with a statistically significant difference (p = 0.004). In our study, the findings indicated a prominent reduction in the number of cases after pharmacist intervention. During the pre-intervention phase, there were 137 cases, representing 37.0% of the CKD population. However, following the intervention by pharmacists, the number decreased significantly to 27 cases, accounting for only 7.3% of the population. This reduction was found to be statistically significant, with a p-value (p = 0.004).

In our study, certain drugs were found to be added for specific indications where they were not initially prescribed. UIs were observed such as anemia, hypertension, gastritis, hyperphosphatemia, and diabetes. Notable examples of such medication additions included such as calcium with vitamin D, omeprazole, prednisolone, erythropoietin, iron supplements such as polymaltose, ferrous sulfate, vitamin B12, antibiotics, metronidazole, metformin, dapagliflozin, and phosphate binders such as calcium acetate. These findings shed light on the practice of modifying medication regimens to address specific needs or conditions that may arise during the course of treatment. These results highlight the positive impact of pharmacist interventions in addressing the identified DRPs. In a study carried out by Adem et al., the pooled estimation of the proportion of DRPs by sub-types revealed that indication-related difficulties (requiring the need for additional medication therapy) were the most often observed DRPs. This reflects the fact that the majority of patients require the start of additional pharmacological therapy for an untreated medical condition or for preventative purposes [28].

It was observed in this study that the patients who were not provided with or did not comprehend vital information about their medication regimens faced an elevated risk of unnecessary medication use. Patients with multiple co-existing conditions commonly take numerous medications simultaneously. Healthcare providers, particularly pharmacists, have a responsibility to prioritize patients with polypharmacy for counseling, simplifying medication regimens, and educating them about the potential hazards of duplicated therapy and drug interactions.

Drugs without indication

Medication without indication was identified in our CKD patients. The prevalence of medications without indication was found in 116 (31.4%) and was reduced to 44 (11.9%) by the intervention of a clinical pharmacist but statistically found not significant (p = 0.072). Examples of such DRPs that were observed to be used without any medical indication by patients included calcium gluconate and non-steroidal anti-inflammatory drugs (NSAIDs) such as diclofenac, which were unnecessary in their treatment.

It is crucial to identify and address these DRPs based on the evidence provided in the literature. By doing so, healthcare professionals can enhance the overall quality of care and ensure the safe and effective use of medications for patients. Continuous assessment and monitoring are essential components of optimizing medication therapy and promoting positive patient outcomes. In a study, it was discovered that DRPs related to drug indications represented in pre-intervention the largest proportion of DRPs (53.5%), among the prescriptions analyzed [30]. In a study during the post-intervention phase, there was a significant decrease in the percentage of prescriptions, exhibiting DRPs related to drug indications, reducing from 53.5% to 28.8% significantly (p < 0.001).

This study concludes that these issues were not a prominent concern for the nephrology doctors involved. The absence of drug treatment despite existing indications may be attributed to various factors, may be due to the doctor prescribing a lower dosage of medication than required by the diagnosis, or the disease being diagnosed but not requiring the use of medication. It is also possible that the patient was already taking medication, and the doctors made a note of it in the diagnosis to avoid prescribing additional drugs. Furthermore, even after the intervention, there was no improvement in prescribing drugs with contraindications. This could be because doctors often prioritize treating the diseases and give less attention to specific contraindications.

Presence of adverse drug reactions

In our study, the presence of adverse reactions was evaluated, and it was found that 90 (24.3%) were observed at the intervention phase and were reduced to cases of 13 (3.5%) with non-significant differences (p = 0.062). As the intervention was not significant, we have observed some of the adverse effects involved in the gastrointestinal system, such as diarrhea from using antibiotics, dry cough because of angiotensin-converting enzyme inhibitors, antidiabetic drug cases, abdominal pain, nausea, bloating, and diarrhea. NSAIDs have additional negative effects, such as reduced potassium excretion leading to hyperkalemia, and reduced sodium excretion resulting in peripheral edema, increased blood pressure, and exacerbation of heart failure.

Hypoglycemia as an adverse reaction to glibenclamide or increasing urinary infection symptoms due to decreased nitrofurantoin efficacy may be especially dangerous in this population. This makes it even more critical and urgent to assess prescriptions for renally excreted medications to ensure that the proper doses are utilized and that additional ADRs are avoided.

Strengths of the study

The study provides a thorough analysis of various DRPs encountered in patients with CKD, covering aspects such as prevalence, contributing factors, and the impact of clinical pharmacist interventions.

The findings are presented clearly, with specific details on the prevalence of different types of DRPs and the effectiveness of pharmacist interventions in addressing them. This clarity enhances the understanding of the study outcomes.

The findings appropriately referenced previous studies to provide context and comparison for the findings, strengthening the credibility of the research.

The study effectively highlights the impact of pharmacist interventions on reducing the occurrence of DRPs, with statistical analysis to support the effectiveness of these interventions.

In this study, practical examples of specific DRPs and interventions were presented, such as the discontinuation of contraindicated medications and adjustment of dosages, which helps illustrate the real-world implications of the findings.

Limitations of the study

The study's findings and discussion are limited to patients with CKD receiving outpatient care in the nephrology department. Therefore, the generalizability of the findings to other patient populations or healthcare settings may be limited.

The discussion does not address potential biases in the study design or data collection process, which could affect the validity of the findings. Acknowledging and addressing these biases would enhance the credibility of the research.

The study could further discuss implications for future research or clinical practice, such as the need for additional studies to validate the effectiveness of pharmacist interventions in different patient populations or settings, or strategies to address persistent challenges in medication management for CKD patients.

## Conclusions

The prevalence of DRPs was high, with an average of 2.903 problems per patient. The prevalence of DRPs among CKD patients was assessed before and after pharmacist intervention. The impact of clinical pharmacist intervention on DRPs was analyzed, revealing a statistically significant reduction in the incidence of improper drug selection, including prescribing the wrong drug, duplication of drugs with similar effects, administration of stoppable drugs, and contraindication of drugs. The incidence of drug interactions, failure to receive medication, poor compliance issues, UIs, and adverse drug reactions also showed a significant decrease post-intervention. The pharmacist intervention had a positive impact on reducing the prevalence of various DRPs among CKD patients, highlighting the crucial role of clinical pharmacists in optimizing medication management for individuals with CKD. Clinical pharmacists played a crucial role in identifying and addressing DRPs, including issues such as improper drug selection, drug interactions, poor compliance, and UIs. The study emphasized the importance of the integration of pharmacist interventions in optimizing medication therapy, reducing drug-related complications, and improving patient outcomes in CKD.
